# TREM1 unleashes immunosuppression in glioma: targeting macrophage polarization as a new therapeutic vulnerability

**DOI:** 10.3389/fimmu.2026.1871988

**Published:** 2026-07-03

**Authors:** Chao Zhang, Da Teng, Chao Wang, Runsheng Feng, Xinqi Huang, Ben Hu, Yu Wang, Ning Lin, Cheng Zhang

**Affiliations:** 1 Department of Neurosurgery, The Affliated Chuzhou Hospital of Anhui Medical University, The First People’s Hospital of Chuzhou, Chuzhou, China; 2 Department of Hepatobiliary Surgery, The First Affiliated Hospital of Anhui Medical University, Hefei, Anhui, China; 3 Graduate School, Bengbu Medical University, Bengbu, China; 4 Science and Technology Innovation Center, Guangzhou University of Chinese Medicine, Guangzhou, China; 5 Department of Disinfection Supply Center, The Affiliated Chuzhou Hospital of Anhui Medical University, Chuzhou, China

**Keywords:** GBM, macrophage, ScRNA-seq, TREM1, tumor microenvironment

## Abstract

**Background:**

Tumor-associated macrophages (TAMs) represent the predominant immune cell subset within the glioma tumor microenvironment (TME). This study aims to investigate the clinical significance of TAM-related TREM1 in glioma and its potential mechanism in promoting malignant progression.

**Methods:**

Bioinformatics analyses were employed to assess the expression pattern and prognostic value of TREM1 in glioma. Single-cell sequencing, immunohistochemistry, and immunofluorescence were used to determine the cellular origin and spatial distribution of TREM1. Using a co-culture model with TREM1 silenced, the potential impact of knocking down TAM-related TREM1 on suppressing malignant progression of glioma and its effect on macrophage polarization markers were evaluated through CCK-8, Transwell, wound healing assays, western blot, and flow cytometry.

**Results:**

TREM1 expression was upregulated in glioma and enriched in subtypes with higher malignant potential, indicating its role as a potential prognostic biomarker. TREM1 was closely associated with M2-type macrophages and promoted their polarization towards the M2 subtype. Knockdown of TREM1 in macrophages reduced the proliferation, invasion, and migration capabilities of glioma cells.

**Conclusion:**

TREM1 serves as a TAM-related oncogenic biomarker that facilitates malignant progression and immune suppression in glioma by promoting M2 macrophage polarization. Targeting TREM1 may offer a novel strategy to enhance the efficacy of immunotherapy for glioma.

## 1 Introduction

Glioblastoma (GBM) is the most aggressive and lethal brain tumor, characterized by significant inter- and intra-tumoral heterogeneity (1, 2). Despite standard treatment involving surgical resection combined with radiotherapy and chemotherapy, the median survival of patients remains poor with no significant improvement (3). In recent years, cancer immunotherapy has demonstrated substantial clinical benefits in various solid tumors; however, its efficacy in GBM remains limited (4). Within the glioma TME, macrophages represent the most abundant immune cell population and are central components of the innate immune system. Beyond tumor cells themselves, TAMs constitute a critical and prominent cellular component in GBM (5, 6). With growing research interest in the TME, the role of immune cells has garnered increasing attention (7). These tumor-infiltrating immune cells not only contribute to an immunosuppressive microenvironment but also play key regulatory roles in tumor progression, metastasis, and therapy resistance (8). Notably, TAMs have been identified as the predominant immune cell subset in GBM (9) and are instrumental in regulating tumor progression and treatment responses (10). During malignant glioma progression, macrophages lose their anti-tumor functions and acquire a tumor-promoting phenotype, thereby facilitating glioma cell proliferation, invasion, and metastasis (11, 12). Consequently, reactivating the anti-tumor functions of macrophages represents a promising therapeutic strategy for glioma treatment (13).

Triggering receptor expressed on myeloid cells-1 (TREM1, also known as CD354) is a key member of the TREM family, which includes TREM1, TREM2, and TREML1 through TREML4 (14). Studies have shown that TREM1 can activate dendritic cells (15) and macrophages (16), and is involved in regulating PD-L1 expression and remodeling the immunosuppressive microenvironment, thereby enhancing immunotherapy efficacy in hepatocellular carcinoma (17). Meanwhile, TREM2 is also widely recognized to be associated with the immunosuppressive functions of TAMs (18). TREM2 expression is upregulated in various tumors, including glioma, and promotes tumor progression and metastasis (19). Although the pathophysiological role of TREM1 was first identified in infectious diseases, growing evidence indicates its involvement in the pathogenesis of various non-communicable diseases (20). TREM1 plays a critical role in inflammation-mediated carcinogenesis (21). Its expression is elevated in solid tumors such as lung cancer, hepatocellular carcinoma, and pancreatic cancer, suggesting its potential as a prognostic biomarker. Furthermore, TREM1-positive TAMs promote intestinal tumorigenesis and are associated with reduced disease-free survival in lung cancer patients (22, 23). In prostate cancer, TREM1 enhances cancer cell motility and invasiveness (24). However, the expression pattern of TREM1 in glioma and its regulatory role in the alternative activation of TAMs remain unclear.

In this study, we investigated the expression pattern of TREM1 in glioma and its role in macrophage polarization and the immunosuppressive TME. By leveraging integrated bioinformatics analysis and an *in vitro* co-culture model, we demonstrated that TREM1 is enriched in more malignant glioma subtypes and is closely associated with inflammatory and immune responses. Further single-cell analysis revealed a strong correlation between TREM1 and M2 macrophages. Functionally, suppression of TREM1 expression effectively inhibited M2 polarization of macrophages and reduced the proliferation, migration, and invasion capabilities of glioma cells. Our findings identify TREM1 as a novel regulatory marker in macrophages, providing new insights into the mechanisms driving malignant progression in GBM and the functional interplay between immune cells and glioma cells.

## 2 Methods

### 2.1 Data acquisition and download

The mRNA-seq data for glioma samples were retrieved and downloaded from The Cancer Genome Atlas (TCGA: http://cancergenome.nih.gov/). Normal tissue data were obtained from the Genotype-Tissue Expression portal (GTEx: (https://www.gtexportal.org/home/). For independent bioinformatic validation, the mRNA-seq-693 and mRNA-seq-325 datasets were acquired from the Chinese Glioma Genome Atlas database (CGGA: http://www.cgga.org.cn/). Patients with incomplete follow-up data were excluded from the subsequent analyses.

### 2.2 Clinical sample acquisition

The clinical samples in this study were primarily used for histological validation via immunohistochemistry and immunofluorescence, including tissue specimens from 5 patients of glioma. All samples were obtained from Chuzhou Hospital Affiliated to Anhui Medical University, and the study was approved by the hospital’s ethics committee, with informed consent obtained from all patients. Inclusion criteria were: pathologically confirmed glioma (WHO grade II-IV) after surgery; no prior radiotherapy, chemotherapy, or immunotherapy; and complete clinicopathological data. Exclusion criteria were: history of other malignant tumors; severe infection or autoimmune diseases; and prior treatment with glucocorticoids or immunomodulatory agents before surgery.

### 2.3 Functional enrichment analysis

To investigate the biological functions of TREM1, we first performed Pearson correlation analysis and separately screened for the top 500 genes significantly positively correlated with TREM1 expression and the top 500 genes significantly negatively correlated with TREM1 expression. The official gene symbols of this gene set were uploaded to the DAVID database (https://davidbioinformatics.nih.gov/), with Homo sapiens specified as the species. Enrichment analyses for Gene Ontology (GO) terms-covering biological processes (BP), molecular functions (MF), and cellular components (CC)-and Kyoto Encyclopedia of Genes and Genomes (KEGG) pathways were subsequently conducted.

### 2.4 Gene set variation analysis

The genes associated with immune and inflammatory processes was retrieved from the AmiGO 2 portal (http://amigo.geneontology.org/amigo). GSVA is a technique employed to assess the variability of a gene set among samples. GSVA analysis can be executed in R through the GSVA package. Functional enrichment scores for the selected gene sets in each glioma sample are evaluated using GSVA, and heatmaps depicting the enrichment results are generated using the pheatmap package. The correlation of the target gene TREM1 with immune or inflammatory processes was ascertained by Pearson correlation analysis.

### 2.5 Gene set enrichment analysis

Based on TREM1 expression levels, samples were stratified into high-expression (≥50%) and low-expression (<50%) groups. The c2.cp.kegg.v7.4.symbols.gmt subset from the Molecular Signatures Database (MSigDB) was used to assess relevant pathways and molecular mechanisms. GSEA was conducted with 1, 000 permutations, and gene sets were restricted to a minimum of 5 and a maximum of 5, 000 genes.

### 2.6 Survival analysis

Survival analysis was performed using the “survival” package in R on datasets from TCGA and CGGA. Kaplan-Meier curves for overall survival (OS) were generated based on target gene expression and survival status. Patients were stratified into “low-expression” and “high-expression” groups using the optimal clinical cutoff value. The p< 0.05 was considered statistically significant.

### 2.7 Single-cell RNA sequencing analyses of TREM1

The scRNA sequencing data from glioma samples were analyzed using datasets obtained from the Tumor Immune Single-Cell Hub 2 database (TISCH2: https://tisch.compbio.cn/) (25). The cellular origin and distribution of TREM1 expression were assessed based on these data. Cell clusters were identified according to established marker genes. TREM1 expression levels across different cell populations were visualized and compared using Uniform Manifold Approximation and Projection (UMAP) plots and violin plots.

### 2.8 Spatial transcriptome analysis

After the preprocessing and quality control of spatial transcriptome data were completed, the normalized expression levels of the target gene in all spatial spots (or cells) were extracted and mapped onto the original coordinate system of the tissue sections. Visualization methods based on spatial location (such as heatmaps or scatter plots) were used to present the continuous expression distribution of the gene across tissue regions.

### 2.9 Cell culture and treatments

The LN229 and U251 glioma cell lines, obtained from ProCell Life Science & Technology Co., Ltd., were cultured in Dulbecco’s Modified Eagle Medium (DMEM) supplemented with 10% fetal bovine serum (FBS). Both cell lines are characterized as isocitrate dehydrogenase (IDH) wild-type. THP-1 cells, sourced from abm Biotech in China, were maintained in RPMI-1640 medium. All cells were cultured at 37°C in a humidified incubator with 5% CO_2_. To differentiate the macrophages, THP-1 cells were first treated with 100 ng/mL phorbol 12-myristate 13-acetate (PMA) for 48 hours to induce an M0-like state. Subsequently, these M0 macrophages were polarized to an M2 phenotype by incubation with 20 ng/mL IL-4 for an additional 48 hours. Additionally, all cell lines were identified to be mycoplasma-free.

### 2.10 Cell viability assay

To assess glioma cell viability, a CCK-8 assay was performed under co-culture conditions. Glioma cells were plated in the lower chambers of a 24-well Transwell system, while M2 macrophages with TREM1 knockdown via siRNA were placed in the upper chambers. Following the manufacturer’s protocol, CCK-8 reagent was added to each well, and the cell culture plates were protected from light and incubated at 37 °C for 2 hours. Absorbance was measured at 450 nm, and the data were subjected to statistical analysis.

### 2.11 Immunohistochemical testing

Immunohistochemical analysis was performed to evaluate the expression of the target proteins in tissue specimens. Briefly, paraffin-embedded tissue sections were deparaffinized and rehydrated using standard procedures. Antigen retrieval was then carried out to expose epitopes. Endogenous peroxidase activity was blocked by incubation with 3% hydrogen peroxide, and nonspecific binding sites were blocked with 5% bovine serum albumin (BSA). The sections were incubated overnight at 4 °C with a primary antibody specific to the target protein. After washing with phosphate-buffered saline (PBS), the sections were incubated with a horseradish peroxidase (HRP)-conjugated secondary antibody for 2 hours at room temperature. Color development was achieved using 3, 3′-diaminobenzidine (DAB) as the chromogen, followed by counterstaining with hematoxylin. The stained sections were then dehydrated through a graded ethanol series, cleared in xylene, and mounted with neutral balsam. Finally, protein expression was examined and imaged under a light microscope.

### 2.12 Immunofluorescence staining

For immunofluorescence staining, prepared tissue sections were fixed with 4% paraformaldehyde (PFA) for 15 minutes and permeabilized with 0.1% Triton X-100 for 15 minutes, followed by three washes with PBS. The sections were then blocked with 5% bovine serum albumin (BSA) at room temperature for 60 minutes and subsequently incubated with the primary antibody at 4°C overnight. The following day, the sections were incubated with a fluorescently conjugated secondary antibody at room temperature for 2 hours. Finally, nuclei were counterstained with DAPI, and images were acquired using a fluorescence microscope.

### 2.13 Western blot analysis

Total protein was isolated from the samples with RIPA lysis buffer. Following extraction, the proteins were resolved by SDS–polyacrylamide gel electrophoresis and electrotransferred onto PVDF membranes. To minimize nonspecific antibody binding, the membranes were blocked for 2 hours at room temperature with 5% non-fat dry milk prepared in TBST (Tris-buffered saline with 0.1% Tween-20). Subsequently, the membranes were exposed to primary antibodies and incubated overnight at 4°C. After thorough washing with TBST, the membranes were treated with horseradish peroxidase (HRP)-labeled secondary antibodies for 2 hours at room temperature. Following another round of TBST washes, the protein bands were detected using an enhanced chemiluminescence (ECL) assay.

### 2.14 Flow cytometry analysis

For flow cytometry analysis, THP-1 cells were polarized into an M2 macrophage phenotype by treatment with 100 ng/mL PMA and 20 ng/mL IL-4. These M2 macrophages were then co-cultured with glioma cells. In parallel, TREM1 expression was knocked down in macrophages using siRNA, followed by a 48h incubation period. After 48h, macrophages were harvested, and the expression of the M2 markers CD163 and CD206 was analyzed by flow cytometry.

### 2.15 Transwell invasion and migration assays

Cell migration and invasion capabilities were assessed by Transwell chamber assays. Uncoated or Matrigel-coated chambers were positioned in 24-well plates. Each upper chamber received 200 μL of serum-free cell suspension, while the lower compartment was filled with 600 μL of complete medium. The plates were then incubated for 24 to 48 hours.

After incubation, non-migratory/invasive cells on the upper surface were carefully wiped away using cotton swabs. The traversed cells on the membrane underside were fixed in 4% formaldehyde for 15 min, rinsed with PBS, and stained with 0.1% crystal violet for another 15 min. Following a final PBS wash, the membrane was air-dried, inverted, and mounted on a microscope slide for imaging and quantitative analysis.

### 2.16 Wound healing assays

Glioma cells in the logarithmic growth phase were plated in 6-well plates and transfected with siRNA using CYTOCH reagent as per the manufacturer’s instructions. Upon reaching approximately 80% confluence, a uniform wound was introduced into the cell monolayer with a sterile 200 μL pipette tip. After PBS washes to remove detached cells, fresh medium was added, and the cultures were returned to the incubator. Wound closure was monitored by imaging the same locations immediately (0 h) and 24 hours after wounding to quantify migration rates.

### 2.17 Statistical analysis

Data analysis was performed using R software and GraphPad Prism. Group comparisons were conducted as follows: a two-tailed Student’s t-test for two groups, one-way ANOVA with Tukey’s *post-hoc* test for multiple groups, and the log-rank test for Kaplan-Meier survival analysis. Statistical significance was defined as p < 0.05, with asterisks indicating specific levels (*p < 0.05, **p < 0.01, ***p < 0.001).

## 3 Result

### 3.1 Potential value of TREM1 in pan-cancer analysis

**Figure 1:**
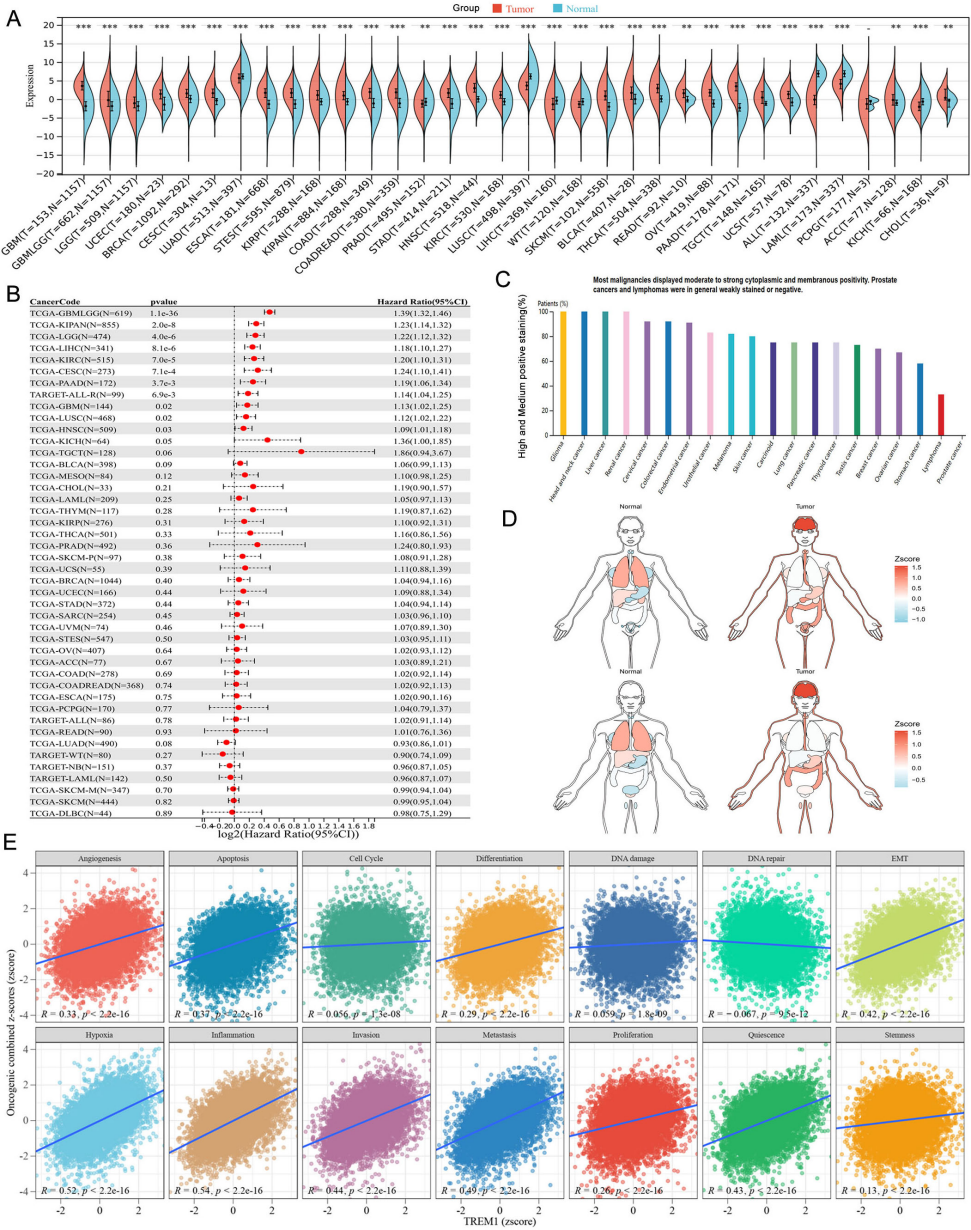
The potential value of TREM1 across pan-cancer. **(A)** Differential expression of TREM1 in pan-cancer, the significance of the difference was tested using the unpaired Wilcoxon rank-sum test. **(B)** Prognostic significance of TREM1 in pan-cancer. **(C)** Immunohistochemical analysis of TREM1 protein expression in human cancers from the HPA database. Staining intensity classification is based on the standard definitions of the Human Protein Atlas (HPA) database. **(D)** Comparison of TREM1 expression between normal and tumor tissues based on the GEPIA database. **(E)** Correlation analysis of the biological functions of TREM1 in pan-cancer. The significance of the difference was tested using an unpaired t-test. *p < 0.05, **p < 0.01, ***p < 0.001.

To investigate the potential value of TREM1, we analyzed its expression across 34 cancer types using data from the TCGA and GTEx databases. The results demonstrated that TREM1 expression was significantly elevated in multiple cancers compared to normal tissues (**Figure 1A**). Furthermore, high TREM1 expression was associated with poor OS in 11 tumor types, including TCGA-GBMLGG (N = 619, p=1.1e-36, HR = 1.39 [1.32-1.46]), TCGA-LGG (N = 474, p=4.0e-6, HR = 1.22 [1.12-1.32]), TCGA-CESC (N = 273, p=7.1e-4, HR = 1.24 [1.10-1.41]), TCGA-KIPAN (N = 855, p=2.0e-8, HR = 1.23 [1.14-1.32]), TCGA-HNSC (N = 509, p=0.03, HR = 1.09 [1.01-1.18]), TCGA-GBM (N = 144, p=0.02, HR = 1.13 [1.02-1.25]), TCGA-KIRC (N = 515, p=7.0e-5, HR = 1.20 [1.10-1.31]), TCGA-LUSC (N = 468, p=0.02, HR = 1.12 [1.02-1.22]), TCGA-LIHC (N = 341, p=8.1e-6, HR = 1.18 [1.10-1.27]), TCGA-PAAD (N = 172, p=3.7e-3, HR = 1.19 [1.06-1.34]), and TARGET-ALL-R (N = 99, p=6.9e-3, HR = 1.14 [1.04-1.25]) (**Figure 1B**). Analysis of the HPA database revealed moderate to strong cytoplasmic and membrane staining of TREM1 protein in most malignancies, whereas weak or negative staining was generally observed in prostate cancer and lymphoma (**Figure 1C**). Additionally, data from the organ expression distribution map further illustrated the differences in TREM1 expression patterns between tumor and normal tissues (**Figure 1D**). Furthermore, gene set enrichment analysis revealed that TREM1 participates in various biological functions in pan-cancer, with its association with inflammation being the most prominent (**Figure 1E**).

### 3.2 TREM1 is enriched in glioma subtypes with higher malignant potential

**Figure 2:**
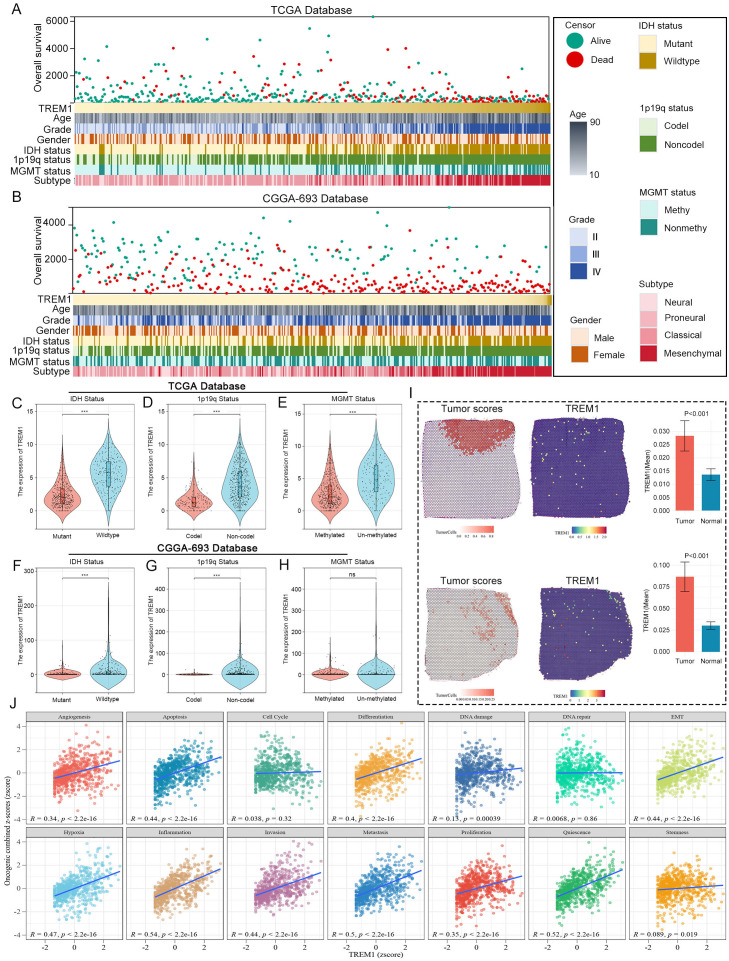
Correlation of TREM1 with Clinical and Molecular Features in Glioma. **(A)** Profile of TREM1 expression and clinical characteristics in the TCGA database. **(B)** Profile of TREM1 expression and clinical characteristics in the CGGA-693 database. **(C, F)** Association between IDH mutation status and TREM1 expression. **(D, G)** Association between 1p/19q co-deletion status and TREM1 expression. **(E, H)** Association between MGMT promoter methylation status and TREM1 expression. **(I)** Spatial transcriptome data revealing the expression pattern of TREM1 in glioma. **(J)** Correlation analysis of the biological functions of TREM1 in glioma. The significance of the difference was tested using an unpaired t-test. ***P<0.001, ns, not significant.

Given the findings from the pan-cancer analysis of TREM1, we focused on investigating its potential role in the malignant progression of glioma. Using bioinformatic approaches, we further examined the expression patterns of TREM1 across various clinical and molecular features of glioma, including IDH mutation status, 1p/19q co-deletion status, and MGMT promoter methylation status. Data from the TCGA and CGGA databases revealed an asymmetric expression distribution of TREM1 across glioma subtypes. Elevated TREM1 expression was associated with shorter OS and was enriched in more aggressive glioma subtypes (**Figures 2A, B**; **Supplementary Figure S1A**). Specifically, in the TCGA database, TREM1 expression was significantly higher in IDH-wildtype patients and in those without 1p/19q co-deletion (**Figures 2C, D**). These findings were validated in the CGGA-693 and CGGA-325 cohorts (**Figures 2F, G**; **Supplementary Figures S1B, C**). Additionally, TREM1 was enriched in patients with unmethylated MGMT promoter, although the difference did not reach statistical significance in the CGGA-693 dataset (**Figures 2E, H**; **Supplementary Figure S1D**). Collectively, these results indicate that elevated TREM1 expression is a characteristic of more malignant glioma subtypes, suggesting its potential involvement in glioma progression, which warrants further investigation. Based on spatial transcriptome data, TREM1 was shown to be highly expressed at glioma tumor sites (**Figure 2I**). Moreover, TREM1 exhibited significant correlations with various biological functions related to the malignant progression of glioma, indicating that TREM1 may serve as a key molecular target for promoting glioma progression (**Figure 2J**).

### 3.3 TREM1 is a specific biomarker for the mesenchymal subtype of glioma

**Figure 3:**
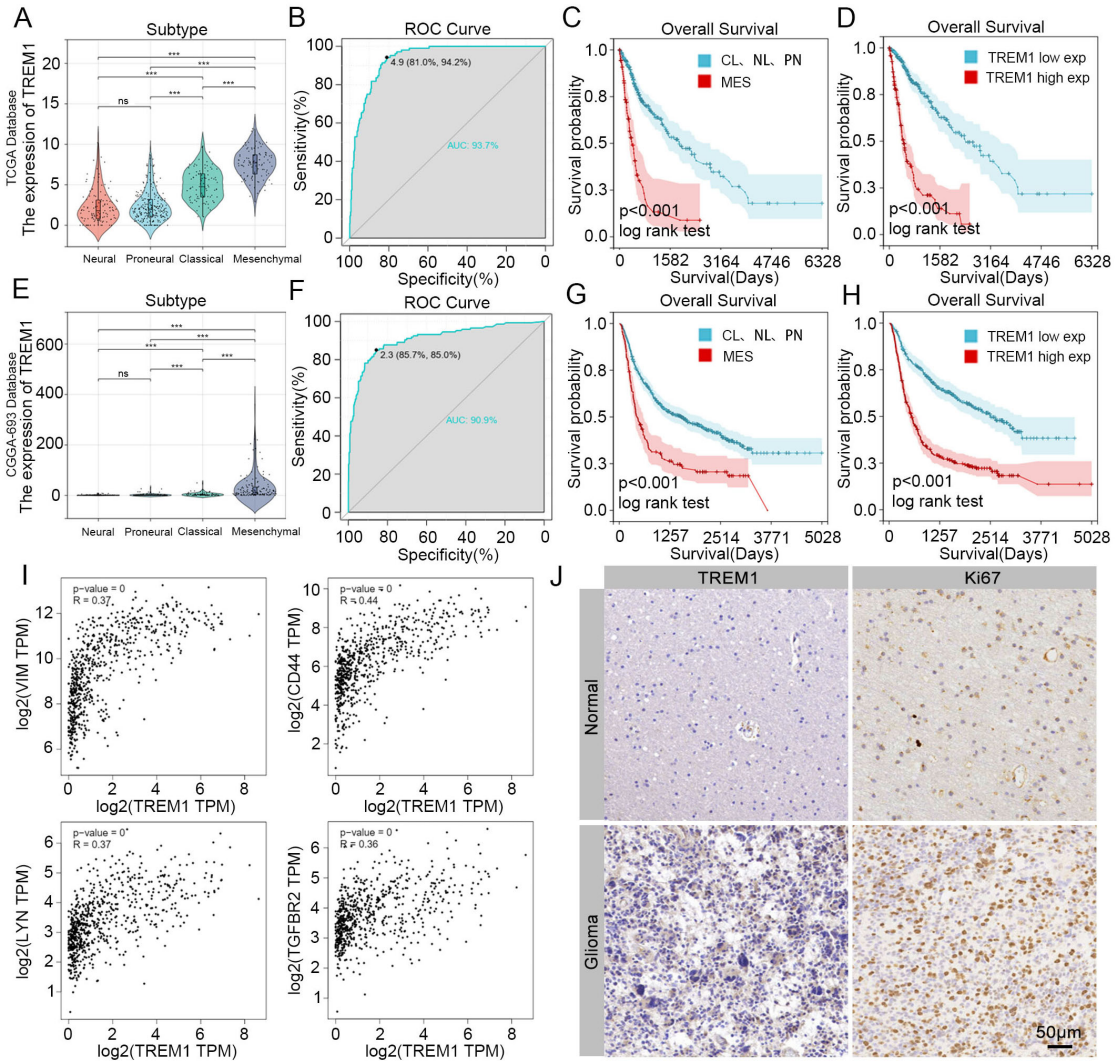
Enrichment of TREM1 in the Mesenchymal Subtype of Glioma. **(A, E)** TREM1 expression across glioma subtypes; **(B, F)** Specific enrichment of TREM1 in the mesenchymal subtype; **(C, G)** Prognosis of the mesenchymal subtype; **(D, H)** Prognostic value of TREM1 expression; **(I)** Correlation of TREM1 with VIM, CD44, LYN, and TGFBP2; **(J)** IHC analysis of TREM1 and KI67 expression in glioma and normal tissues. Brown signals represent positive expression of TREM1 or Ki67, while blue signals from hematoxylin counterstaining represent cell nuclei. Significance of differences was tested using ANOVA and log rank test. ***P<0.001, ns, not significant.

Comprehensive molecular classification of glioma has enhanced prognostic prediction, prompting us to profile TREM1 expression across four major subtypes (26). Analysis of the TCGA and CGGA datasets consistently revealed that TREM1 was robustly enriched in the mesenchymal subtype relative to others (**Figures 3A, E**; **Supplementary Figure S2A**). We further assessed the diagnostic potential of TREM1 using ROC curve analysis, which yielded high AUC values of 0.937 in the TCGA(**Figure 3B**) datasets and 0.909/0.925 in the CGGA cohorts (**Figure 3F**; **Supplementary Figure S2B**). These results strongly suggest TREM1 as a potential biomarker for the mesenchymal subtype in glioma.

Furthermore, Kaplan–Meier analysis showed that patients with the mesenchymal subtype had significantly poorer prognosis (**Figures 3C, G**, **Supplementary Figure S2C**). Similarly, elevated TREM1 expression was also associated with worse clinical outcomes (**Figures 3D, H**, **Supplementary Figure S2D**). Further analysis confirmed the prognostic value of TREM1 in GBM patients (**Supplementary Figure 3**). Furthermore, the results demonstrated that TREM1 expression was significantly correlated with stemness-associated genes (such as CD44 and VIM) and mesenchymal transition-related genes (including LYN and TGFBR2) (2, 27) (**Figure 3I**). The immunohistochemical staining results showed that TREM1 expression was significantly elevated in glioma tissues compared with normal brain tissues, and the proportion of Ki67-positive cells was also significantly increased. This indicates that areas with high TREM1 expression are often accompanied by higher levels of Ki67 staining signals, suggesting that TREM1 expression is associated with increased proliferative activity of glioma cells (**Figure 3J**).

### 3.4 TREM1 found to be strongly associated with immunity and inflammation in glioma patients

**Figure 4:**
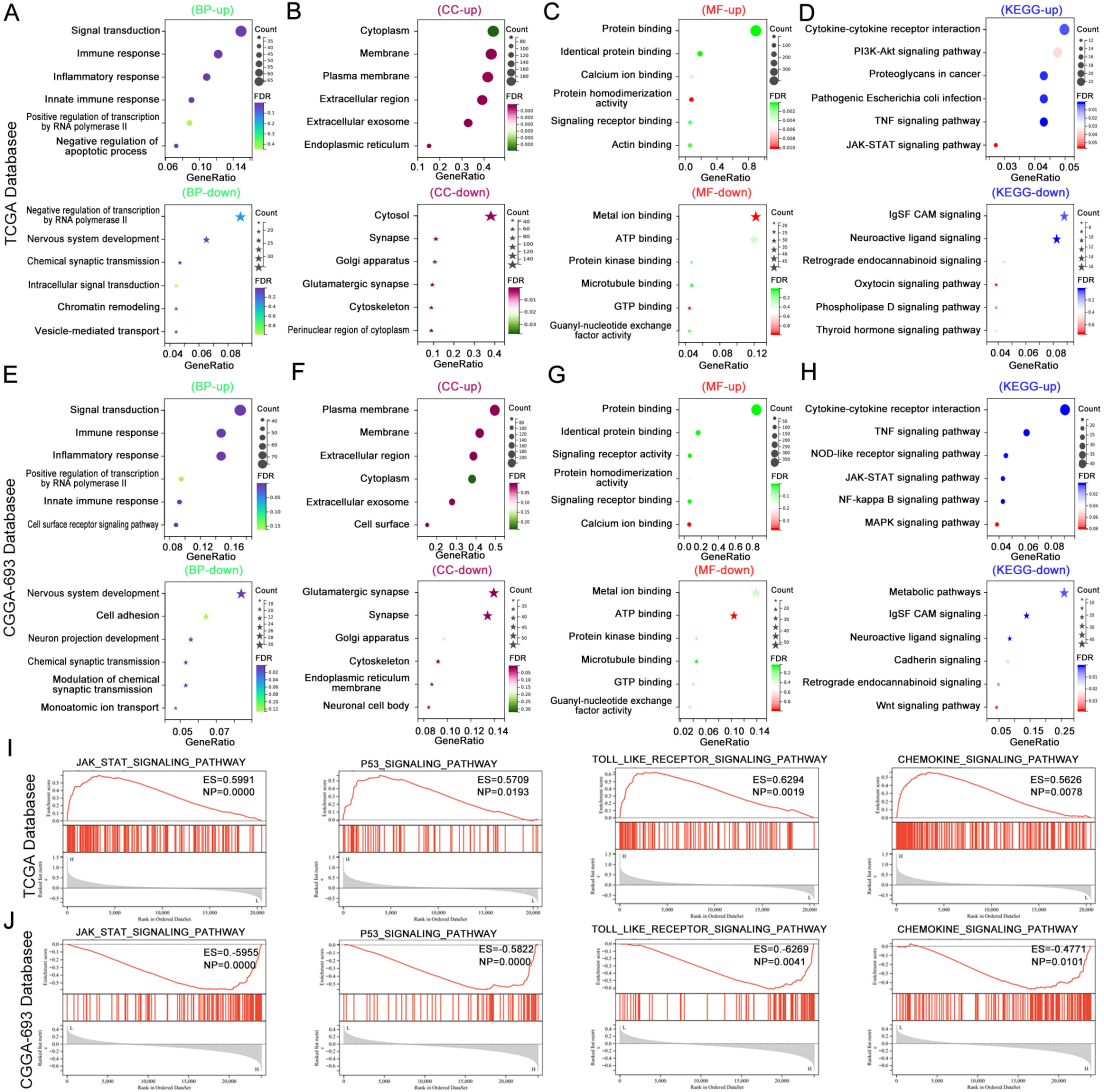
GO and KEGG functional enrichment analysis of TREM1-related genes. **(A, E)** GO analysis results for TREM1 co-expressed genes in BP; **(B, F)** GO analysis results for TREM1 co-expressed genes in CC; **(C, G)** GO analysis results for TREM1 co-expressed genes in MF; **(D, H)** KEGG analysis results for TREM1 co-expressed genes; **(I, J)** GSEA analysis of TREM1. BP, Biological Process; CC, Cellular Component; MF, Molecular Function.

Our existing studies have revealed the prognostic value of TREM1 in glioma cells. To further elucidate the specific functional mechanisms by which TREM1 mediates the poor prognosis of glioma cells, we performed Pearson correlation analysis to separately screen for the top 500 genes significantly positively correlated and the top 500 genes significantly negatively correlated with TREM1 expression in the TCGA database (P<0.05). Subsequently, GO and KEGG enrichment analyses were performed on these two gene sets separately. For the positively correlated genes, the results showed that their biological processes (BP) were mainly concentrated in Signal transduction, Immune response, and Inflammatory response (**Figure 4A**); the cellular components (CC) were predominantly enriched in the Cytoplasm (**Figure 4B**); the molecular functions (MF) were primarily related to Protein binding (**Figure 4C**); KEGG pathway analysis revealed that the positively correlated genes were mainly involved in pathways such as the JAK-STAT signaling pathway (**Figure 4D**). These results suggest that TREM1 may promote malignant progression in glioma by activating immune- and inflammation-related pathways. For the negatively correlated genes, enrichment analysis showed that they were mainly involved in biological processes such as Nervous system development and Chromatin remodeling (**Figure 4A**); the cellular components were predominantly enriched in the Cytosol (**Figure 4B**); the molecular functions were mainly related to Metal ion binding (**Figure 4C**); KEGG pathway analysis indicated that the negatively correlated genes were primarily involved in pathways such as IgSF CAM signaling (**Figure 4D**). Moreover, similar results were obtained in the CGGA-693 and CGGA-325 databases (**Figures 4E–H**; **Supplementary Figures 4A-D**). Furthermore, further GSEA analysis revealed that pathways such as the JAK-STAT signaling pathway, p53 signaling pathway, Toll-like receptor signaling pathway, and chemokine signaling pathway were activated in the high TREM1 expression group, suggesting that they may be involved in TREM1-mediated poor prognosis in glioma (**Figures 4I, J**, **Supplementary Figure 4E**).

### 3.5 Immunity and inflammation within the TME: a potential specific mechanism by which TREM1 mediates poor prognosis in glioma patients

**Figure 5:**
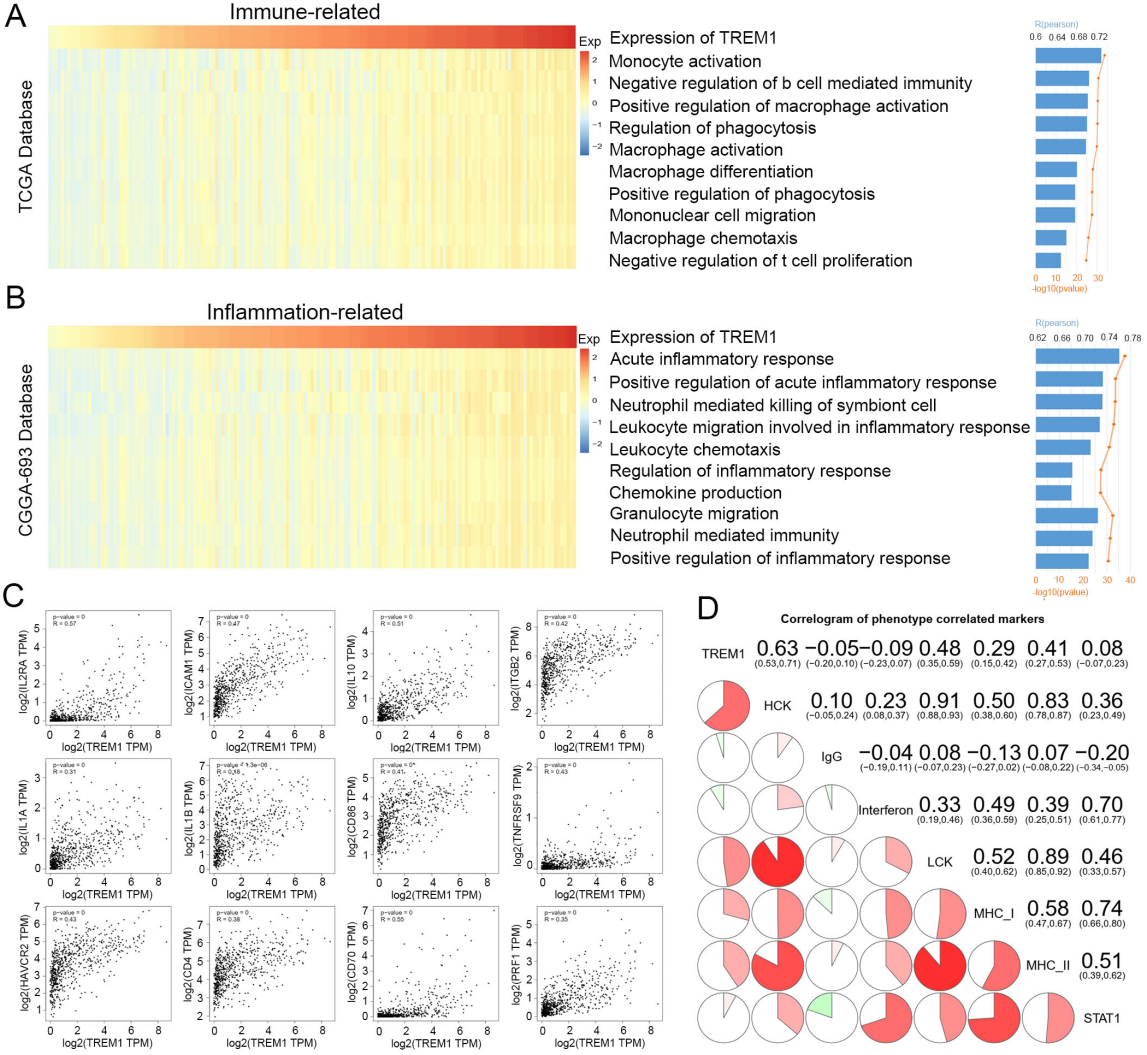
TREM1-Associated Immune and Inflammatory Functions in glioma Patients. **(A)** GSVA analysis of immune-associated functions in glioma patients; **(B)** GSVA analysis of inflammation-associated functions in glioma patients; **(C)** Correlation analysis between TREM1 and immune checkpoints; **(D)** A correlation matrix plot illustrating the relationship between TREM1 and inflammatory gene clusters.

The TME plays a critical role in the growth and metastasis of many tumors, including gliomas, and influences both the survival and therapeutic strategies of glioma patients. In light of the functional enrichment analysis results of TREM1 co-expressed genes, we hypothesized that TREM1 may mediate the developmental evolution of GBM patients through immune and inflammatory mechanisms. Thus, we demonstrated that the expression level of TREM1 was closely associated with numerous immune and inflammatory gene sets using GSVA analysis, particularly with the functions of microglia and macrophages (**Figures 5A, B**). Based on these findings, we speculated that TREM1 could impact the prognosis of glioma patients by affecting the level of immune infiltration. Consequently, we evaluated the relationship between TREM1 and glioma immune infiltration using the ESTIMATE algorithm. We observed that TREM1 expression was positively correlated with the stromal score (R = 0.60, **Supplementary Figure 5C**), immune score (R = 0.59, **Supplementary Figure 5D**), and ESTIMATE score (R = 0.62, **Supplementary Figure 5E**), with similar results obtained in LGG patients or all glioma patients. We then assessed each patient’s aDC, B-cells, CD8+_T-cells, Macrophages, Macrophages_M1, Macrophages_M2, Tregs, ImmuneScore, StromaScore, and MicroenvironmentScore infiltration scores, among others, based on TREM1 gene expression using the R package IOBR’s deconvo_xCell method (28) (**Supplementary Figure 5A**). Additionally, we further compared the correlation of TREM1 with macrophages (R = 0.63), M1 macrophages (R = 0.65), and M2 macrophages (R = 0.41) in GBM (**Supplementary Figure 5B**).

Based on the aforementioned findings, TREM1 is likely to be a key factor contributing to immunosuppression in GBM patients. Consequently, we analyzed the correlation between TREM1 expression and 21 common immune checkpoints, including IL2RA, CD4, and CD47. The results showed that TREM1 was significantly positively correlated with most immune checkpoint molecules. The co-expression of TREM1 with multiple immune checkpoints suggests that glioma patients with high TREM1 expression may be in a stronger immunosuppressive state and exhibit poor response to existing immune checkpoint inhibitors (**Figure 5C**, **Supplementary Figure S6**). Given that immunity and inflammation are inextricably linked, and pro-tumor inflammation in GBM patients is likely to mediate their poor prognosis, we explored the role of TREM1 in inflammation using the seven macrogenes defined earlier (29). We observed that TREM1 expression was positively correlated with HCK, interferon, LCK, MHC-I, MHC-II, and STAT1 metabolism, but negatively correlated with IgG metabolism in GBM patients (**Figure 5D**). Overall, TREM1 appears to play an important role in the immunobiological processes of GBM patients.

### 3.6 TREM1 was predominantly expressed in M2-like tumor-associated macrophages in GBM

**Figure 6:**
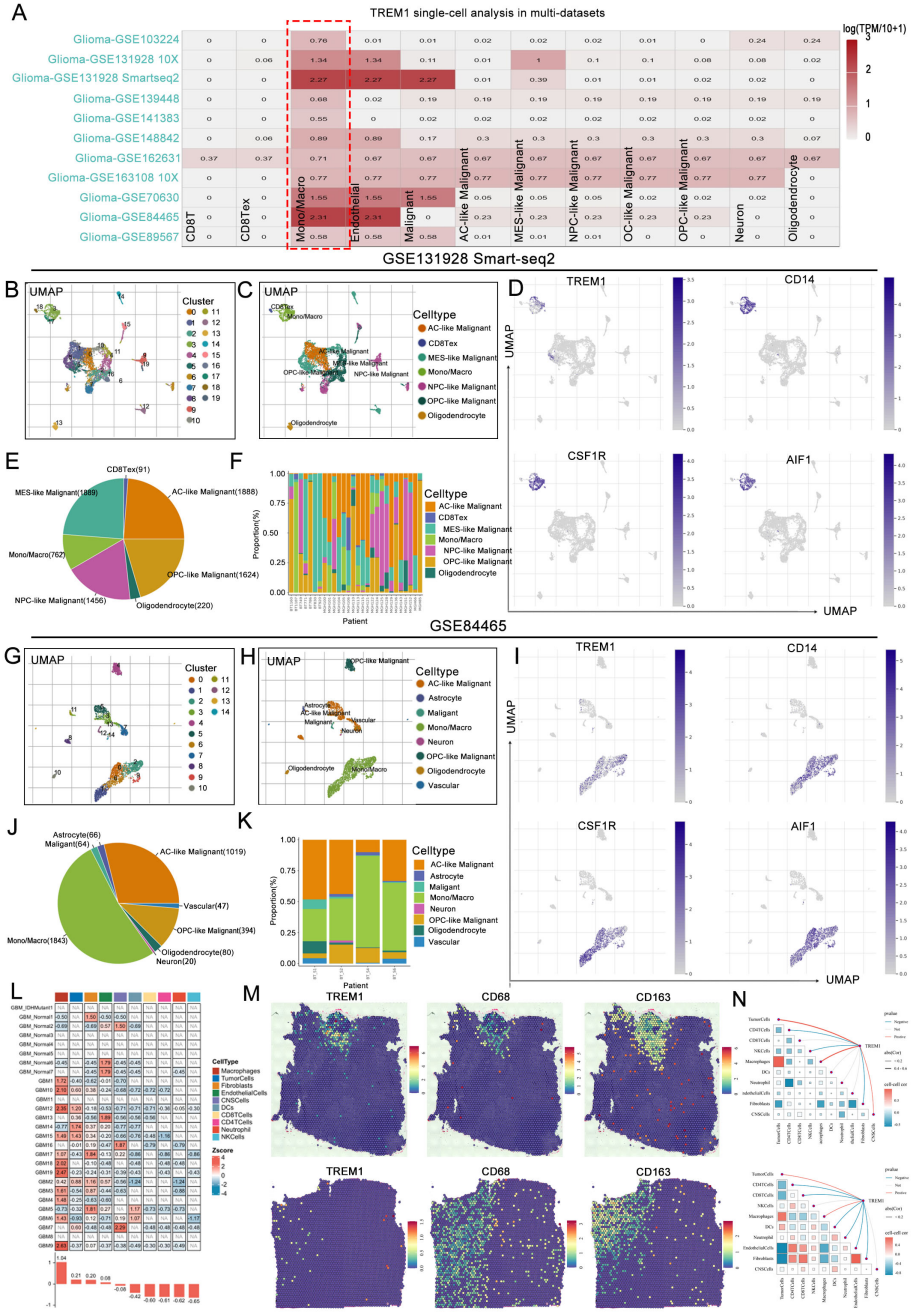
TREM1 is Enriched in M2-like TAMs in Glioma. **(A)** Analysis of TREM1 expression patterns across multiple single-cell datasets; **(B, G)** UMAP plots visualizing the dimensionality reduction and clustering results; **(C, H)** UMAP plots showing the annotated cell types; **(D, I)** UMAP plots displaying the distribution of TREM1 expression; **(E, J)** Pie charts illustrating the compositional distribution of cell subtypes; **(F, K)** Bar plots showing the distribution of cell subtypes in patients. **(L)** Expression pattern of TREM1 in glioma spatial transcriptome data. **(M)** Colocalization analysis of TREM1 and M2-TAMs in glioma. N, Intercellular correlation analysis of TREM1 in glioma.

Given the remarkable immunomodulatory capacity of TREM1, we further investigated its cellular origin using a single-cell atlas. A comprehensive single-cell transcriptomic analysis was performed across multiple glioma datasets. Results revealed that TREM1 was predominantly enriched in cell subpopulations annotated as Mono/Macro, with particularly high expression levels observed in the GSE131928 Smartseq2 and GSE84465 datasets (**Figure 6A**). These two datasets were therefore selected for in-depth analysis.

In the GSE131928 Smartseq2 dataset, we identified multiple cellular subpopulations, including AC-like Malignant, MES-like Malignant, NPC-like Malignant, OPC-like Malignant, CD8Tex, Oligodendrocytes, and Mono/Macro (**Figures 6B, C**). The GSE84465 dataset contained AC-like Malignant, OPC-like Malignant, Astrocytes, Malignant cells, Neurons, Oligodendrocytes, Vascular cells, and Mono/Macro (**Figures 6G, H**). Tumor cells were identified based on high expression of PTPRZ1, NFIB, and SOX2 (**Supplementary Figures 7A, B**), while TAMs were defined by elevated expression of CD14, CSF1R, and AIF1. Notably, TREM1 expression closely aligned with established TAM markers, indicating its enrichment in macrophages and suggesting a potential immunomodulatory role through this cell type (**Figures 6D, I**; **Supplementary Figures S8A, B**). Consistent with previous studies, Mono/Macro constituted a substantial proportion of cells in the GSE131928 Smart-seq2 dataset, underscoring their importance in remodeling the TME (**Figures 6E, F**). Similar observations were made in the GSE84465 dataset (**Figures 6J, K**). Further analysis revealed co-expression of TREM1 with the M2 macrophage marker CD163 (**Supplementary Figures 9A, B**). In light of the established significance of TREM2 in TAMs (30), we also identified a consistent expression pattern between TREM1 and TREM2 (**Supplementary Figures 9A, B**, **S10**). Additionally, spatial transcriptome data showed that TREM1 is enriched in glioma macrophages (**Figure 6L**), shares a similar spatial expression pattern with M2-TAMs (**Figure 6M**), and intercellular correlation analysis revealed that TREM1 is most closely linked to tumor cells and macrophages in glioma (**Figure 6N**). Collectively, these findings suggest that TREM1 may function as a TAM-associated biomarker with potential immunomodulatory roles in glioma.

### 3.7 TREM1 promotes glioma proliferation, migration, and invasion through M2-like TAM-mediated mechanisms

**Figure 7:**
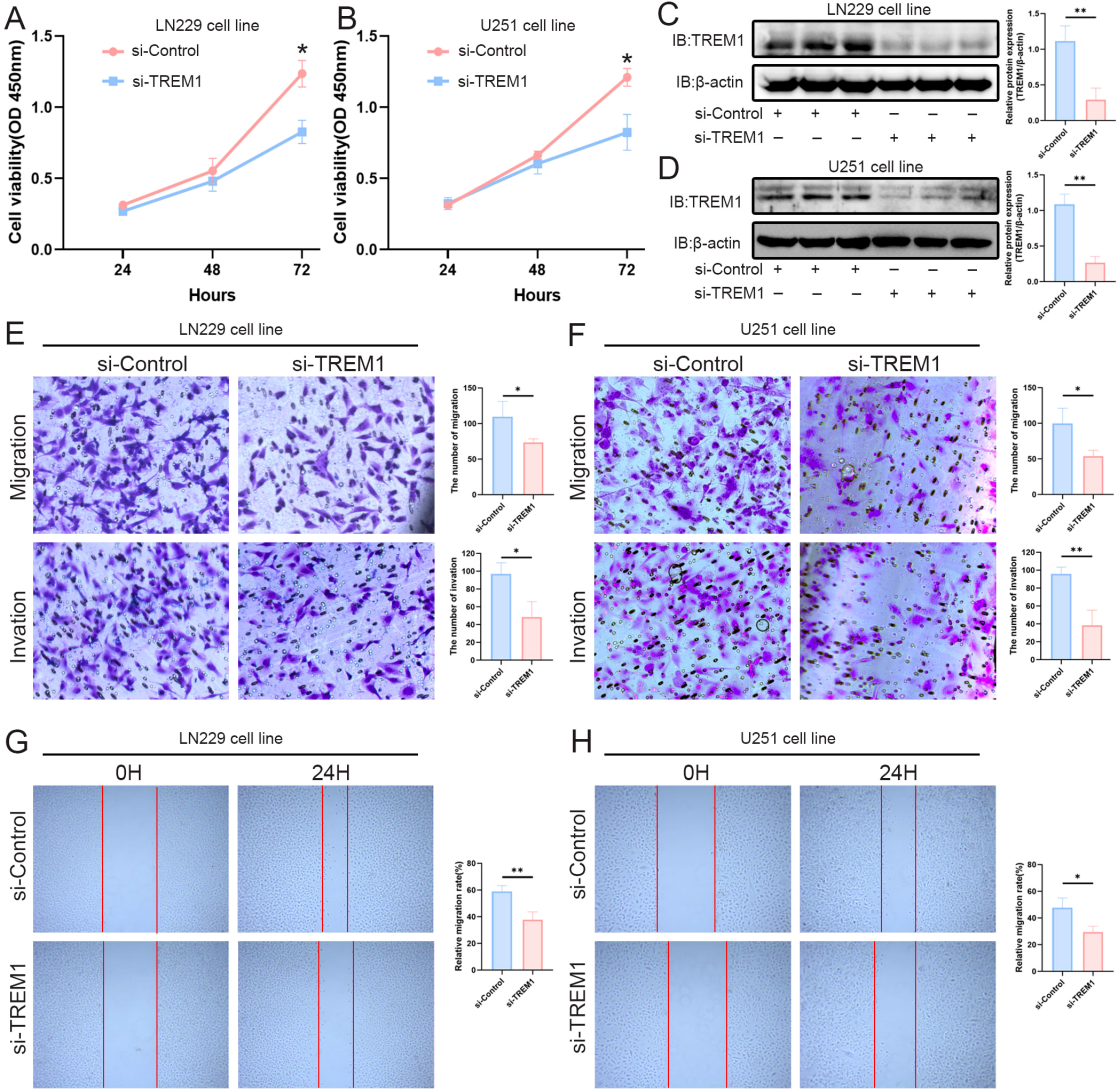
TREM1 Promotes Malignant Progression of Glioma. **(A, B)** CCK-8 assay assessing the effect of TREM1 knockdown in macrophages on glioma cell viability; **(C, D)** Western blot analysis validating the efficiency of TREM1 knockdown in macrophages in the co-culture model; **(E, F)** Transwell assays evaluating the effects of TREM1 knockdown in macrophages on the migration and invasion capabilities of glioma cells; **(G, H)** Wound healing assay detecting the effect of TREM1 knockdown in macrophages on glioma cell migration. *P<0.05 *P<0.05*, **P<0.01 *P<0.01*.

To further elucidate the potential role of TREM1 in M2 macrophages during glioma malignant progression, we established a co-culture system to investigate its specific effects on glioma cells. CCK-8 assays demonstrated that knockdown of TREM1 in M2 macrophages significantly attenuated the proliferation rates of both LN229 and U251 cells (**Figures 7A, B**). Western blot analysis confirmed efficient TREM1 knockdown in macrophages (**Figures 7C, D**). Transwell assays further revealed that TREM1 silencing in macrophages markedly reduced the migration and invasion capabilities of glioma cells (**Figures 7E, F**). Consistent with these findings, wound healing assays yielded similar results, showing impaired cell motility upon TREM1 depletion (**Figures 7G, H**).

### 3.8 Lowering TREM1 exerts anti-tumor effects by inhibiting M2 polarization of macrophages

**Figure 8:**
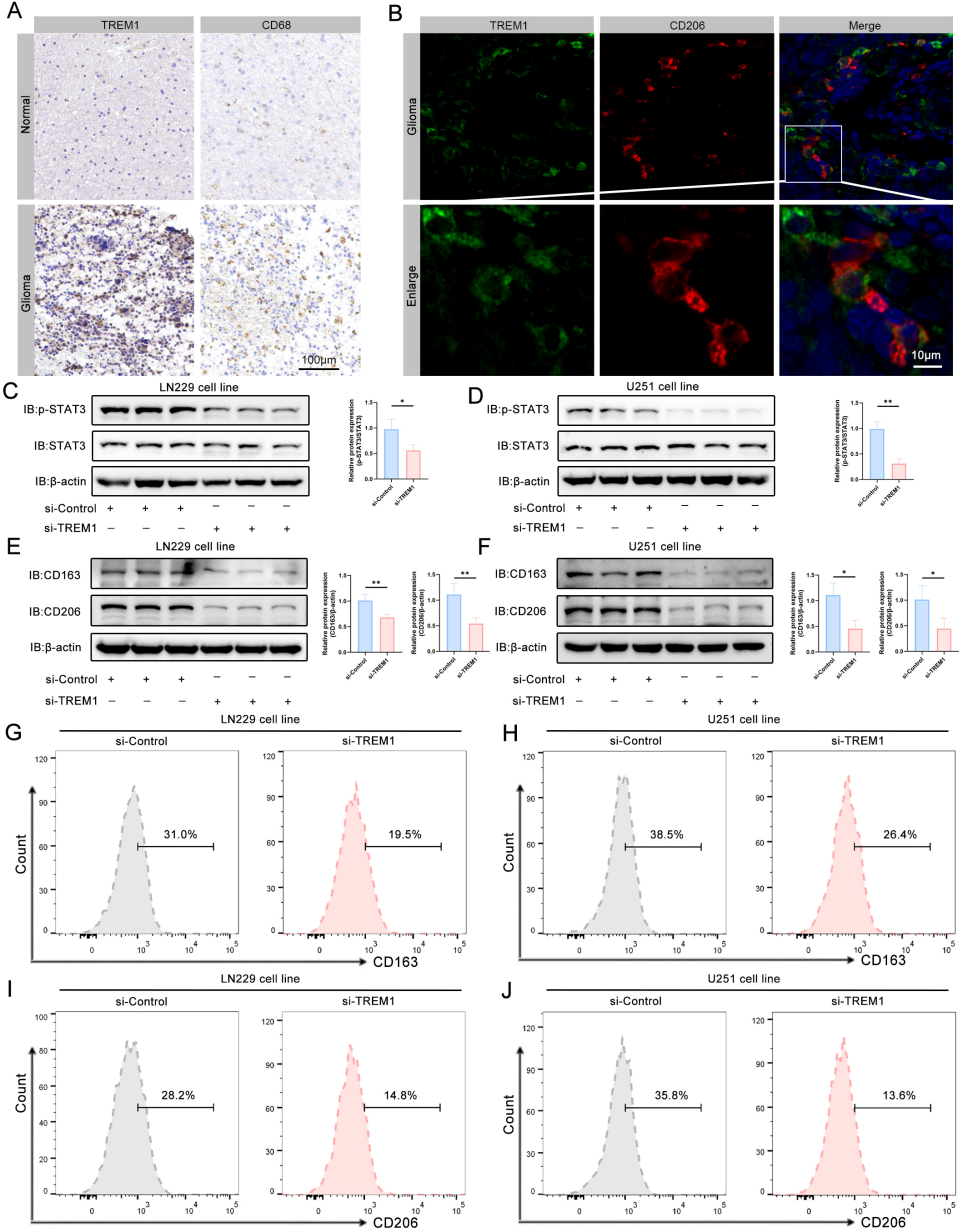
TREM1 influences the glioma tumor microenvironment by promoting macrophage M2 polarization. **(A)** IHC analysis of TREM1 and CD68 expression in glioma tissues; **(B)** Immunofluorescence staining showing the co-localization of TREM1 and CD206 in glioma specimens; **(C, D)** Western blot analysis of p-STAT3 and STAT3 expression levels; **(E, F)** Western blot analysis of CD163 and CD206 expression levels; **(G, H)** Flow cytometric analysis of CD163 expression; **(I, J)** Flow cytometric analysis of CD206 expression. *P<0.05 *P<0.05*, **P<0.01 *P<0.01*.

Given that knockdown of TREM1 significantly inhibits the invasion and migration of glioma cells through macrophages, we hypothesized that TREM1 may exert anti-tumor effects by regulating the polarization state of macrophages. Therefore, we performed immunohistochemical analysis in glioma, and the results showed that high expression of TREM1 in glioma samples was accompanied by high expression of CD68, suggesting a potential association between macrophage infiltration and TREM1 in glioma (**Figure 8A**). The immunofluorescence co-localization analysis in this figure showed that TREM1 and the M2 macrophage marker CD206 were co-expressed in glioma tissues. This finding further validates the enrichment of TREM1 in M2-type TAMs, indicating that TREM1 may regulate the glioma immune microenvironment by promoting M2 polarization. This result directly confirms at the protein level that TREM1 is mainly expressed in M2-type TAMs, consistent with the single-cell transcriptome and spatial transcriptome analyses, and further supports TREM1 as a functional marker of M2-type TAMs involved in maintaining the immunosuppressive microenvironment (**Figure 8B**). Based on the results of TREM1-related functional enrichment analysis, we hypothesized that TREM1 may regulate macrophages through the JAK/STAT signaling pathway. Among the JAK-STAT pathways, STAT3 in particular has been widely reported as a core signaling axis driving M2 polarization of macrophages. Following this clue, we further performed validation experiments in an *in vitro* co-culture system. The results showed that knockdown of TREM1 inhibited the phosphorylation level of STAT3 (**Figures 8C, D**). Combining single-cell transcriptome analysis, spatial transcriptome analysis, and immunohistological findings, we hypothesize that TREM1 may contribute to the establishment of a tumor-favorable immune microenvironment by promoting the recruitment of M2-type macrophages and maintaining their immunosuppressive phenotype. To further investigate the role of TREM1 in macrophage polarization, we established co-culture models. In the THP-1 and LN229 co-culture system, siRNA-mediated knockdown of TREM1 in macrophages led to downregulation of the M2 markers CD206 and CD163, as confirmed by western blot (**Figure 8E**). Consistent with this, flow cytometry analysis showed reduced protein levels of CD206 and CD163 upon TREM1 knockdown (**Figures 8G, I**). Similar results were observed in the THP-1 and U251 co-culture model (**Figures 8F, H, J**). These findings suggest that TREM1 serves as a novel M2 macrophage marker, and knockdown of TREM1 can reduce M2 polarization of macrophages, thereby remodeling the immunosuppressive TME of glioma to exert anti-tumor effects.

## 4 Discussion

GBM is the most common and aggressive primary central nervous system tumor. It poses a major challenge in oncology due to its complex pathogenesis, highly invasive behavior, and resistance to conventional therapies such as temozolomide (7). Current research on the TME has primarily focused on the role of immune cells in glioma progression. TAMs represent a major cellular component in the glioma TME and significantly contribute to malignant progression, particularly through the establishment of an immunosuppressive TME (31). A higher density of TAMs within the tumor is often associated with poorer prognosis in glioma patients (32). Therefore, elucidating the mechanisms by which TAMs promote glioma progression is crucial for improving the efficacy of immunotherapeutic strategies in glioma treatment.

Myeloid immune checkpoints are receptors expressed on myeloid cells that suppress their activity and contribute to tumor immune evasion (33). Although current cancer immunotherapies, particularly immune checkpoint blockade, have significantly improved long-term outcomes in various cancers and achieved remarkable success in numerous solid tumors (34). Despite considerable advances in targeting immune checkpoints, response rates among glioma patients remain unsatisfactory (1, 30, 35). These disappointing outcomes motivate the continued search for additional immune checkpoints to optimize immunotherapy strategies for GBM. In our study, TREM1 was identified as an immune-related biomarker in glioma. Mechanistically, TREM1 is enriched in glioma-associated macrophages, and its knockdown was found to inhibit M2 polarization of macrophages, thereby modulating the immunosuppressive TME and enhancing anti-tumor immunity. The glioma microenvironment itself tends to promote an M2 phenotype. TREM1 is mainly enriched in M2-type TAMs in the glioma microenvironment and maintains its immunosuppressive function by regulating M2 polarization-related genes. Future studies could systematically dissect the potential impact of TREM1 on M1 polarization using *in vitro* models of induced M1/M2 polarization.

Single-cell analysis has become a vital tool for dissecting tumor composition and plays a pivotal role in revealing cellular heterogeneity and the TME (36). In this study, TREM1 was identified as a potential target for immunotherapy in glioma patients. Previous studies have shown that TREM1 inhibitors can suppress tumor growth and enhance the anti-tumor efficacy of PD-L1 blockade (17); however, in-depth investigations of TREM1 in glioma remain limited. By integrating large-scale single-cell RNA sequencing data with bulk RNA sequencing data, we identified TREM1 as a specific marker for M2 macrophages in the glioma TME. High TREM1 expression is enriched in high-risk glioma subtypes characterized by IDH-wildtype, non-1p/19q co-deleted, and MGMT unmethylated status, consistent with our single-cell and co-culture experiments showing that TREM1 promotes tumor proliferation, migration, and immunosuppression through M2 macrophage polarization. Therefore, TREM1 not only serves as a molecular marker of malignant glioma but may also influence patient prognosis and response to immunotherapy/chemotherapy.

Further functional enrichment analysis revealed that TREM1 plays a key role in regulating immune and inflammation-related mechanisms. Currently, immune checkpoint blockade therapy still faces challenges in the clinical treatment of glioma patients (37, 38). We found that TREM1 exhibits strong synergistic effects with most known immune checkpoint molecules (such as IL2RA, ICAM1, ITGB2, CD44, and CD47). Evidence indicates that TREM1 inhibitors can enhance the immune efficacy of anti-PD-L1 therapy in hepatocellular carcinoma patients (17). Moreover, combined blockade of PD-1 and CSF-1R in glioma patients leads to more sustained anti-tumor effects (39). Therefore, although TREM1 inhibitors have not yet been approved for clinical use, combination therapy undoubtedly represents a future direction for glioma treatment (40, 41). The function of TREM1 in immune-inflammatory responses and its synergistic relationship with multiple immune checkpoints open new perspectives for the treatment of glioma patients.

TREM1, an emerging inflammatory receptor, has been reported to play a role in inflammation and immune responses within the TME (42, 43). Given that inflammasome-dependent cytokine and antigen release can activate, shape, or suppress adaptive immunity (44), our study reveals a specific role for TREM1 in TAMs of glioma patients. As crucial antigen-presenting cells, macrophages not only participate in the regulation of T cell function but also secrete factors—depending on their M1/M2 polarization—that modulate T cell responses (45). Previous studies have shown that TREM1 is associated with M2 macrophage polarization markers (46) and may mediate macrophage polarization in hepatocellular carcinoma via the PI3K/AKT pathway (47). Our findings further confirm that TREM1 is specifically expressed in M2 macrophages and is significantly correlated with the extent of macrophage infiltration in glioma. Knockdown of TREM1 suppressed M2 polarization of macrophages, thereby inhibiting the proliferation, invasion, and migration capabilities of glioma cells. Therefore, we propose that targeting TREM1 may improve TAM reprogramming and promote TME remodeling, ultimately enhancing the OS of glioma patients.

Although this study confirms TREM1 as a reliable biomarker in glioma patients, its immunotherapeutic relevance needs to be further validated in dedicated immunotherapy cohorts. The mechanistic relationships between TREM1 and distinct TAM subpopulations remain to be systematically investigated, and detailed experimental verification using *in situ* tumor models will be an important focus of future work. The clinical samples in this study were mainly used for histological validation. The sample size was relatively limited, and complete follow-up information was lacking; therefore, we were unable to further evaluate the association between TREM1 protein expression and patient clinicopathological characteristics or prognosis. Future validation based on large-scale, multi-center clinical cohorts is warranted. In summary, our findings identify TREM1 as a potential biomarker in glioma-associated macrophages, demonstrate that its knockdown suppresses M2 polarization, and highlight its targeting as a promising strategy for improving immunotherapy outcomes in glioma patients.

## 5 Conclusion

Through integrated analysis of single-cell RNA sequencing and bulk RNA sequencing data, we have established TREM1 as a biomarker for TAMs in the TME of glioma patients. TREM1 is enriched in more aggressive glioma subtypes and promotes glioma progression by mediating immunosuppression through TAMs. Knockdown of TREM1 suppresses M2 macrophage polarization, thereby exerting anti-tumor effects and revealing a potential therapeutic strategy to enhance immunotherapy outcomes in glioma.

## Data Availability

The original contributions presented in the study are included in the article/**Supplementary Material**. Further inquiries can be directed to the corresponding authors.
