# Supplementary material for: TREM1 unleashes immunosuppression in glioma: targeting macrophage polarization as a new therapeutic vulnerability
Source: Front Immunol. 2026 Jul 3;17:1871988. doi: 10.3389/fimmu.2026.1871988 (PMC13376122; doi:10.3389/fimmu.2026.1871988)
Supplement: Supplementary file 1 [file Table1.docx]

**Supplementary documents**

**Title Page**

**Article title:**

TREM1 Unleashes Immunosuppression in Glioma: Targeting Macrophage Polarization as a New Therapeutic Vulnerability

**Authors’ names:**

Chao Zhang^1^, Da Teng^2^, Chao Wang^1^, Runsheng Feng^3,1^, Xinqi Huang^1^, Ben Hu^4^, Yu Wang^1^*,Ning Lin^1^*, Cheng Zhang^5^*

**Authors’ affiliations:**

^1^Department of Neurosurgery, The Affliated Chuzhou Hospital of Anhui Medical University, The First People's Hospital of Chuzhou, Chuzhou 239000, China.

^2^Department of Hepatobiliary Surgery, The First Affiliated Hospital of Anhui Medical University, Hefei, Anhui 230001, China.

^3^Graduate School, Bengbu Medical University, Bengbu 233000, China.

^4^Science and Technology Innovation Center, Guangzhou University of Chinese Medicine, Guangzhou 510405, China.

^5^Department of Disinfection Supply Center, The Affiliated Chuzhou Hospital of Anhui Medical University, Chuzhou, 239000, China.

Chao Zhang and Da Teng contributed equally to this work.

**Corresponding author:**

Cheng Zhang: [15755088880@163.com](mailto:zll3526029@163.com)

Ning Lin: [lin2007512@vip.163.com](mailto:linning@ahmu.edu.cn)

Yu Wang: wangyu6517@126.com

**Supplementary Figure S1**


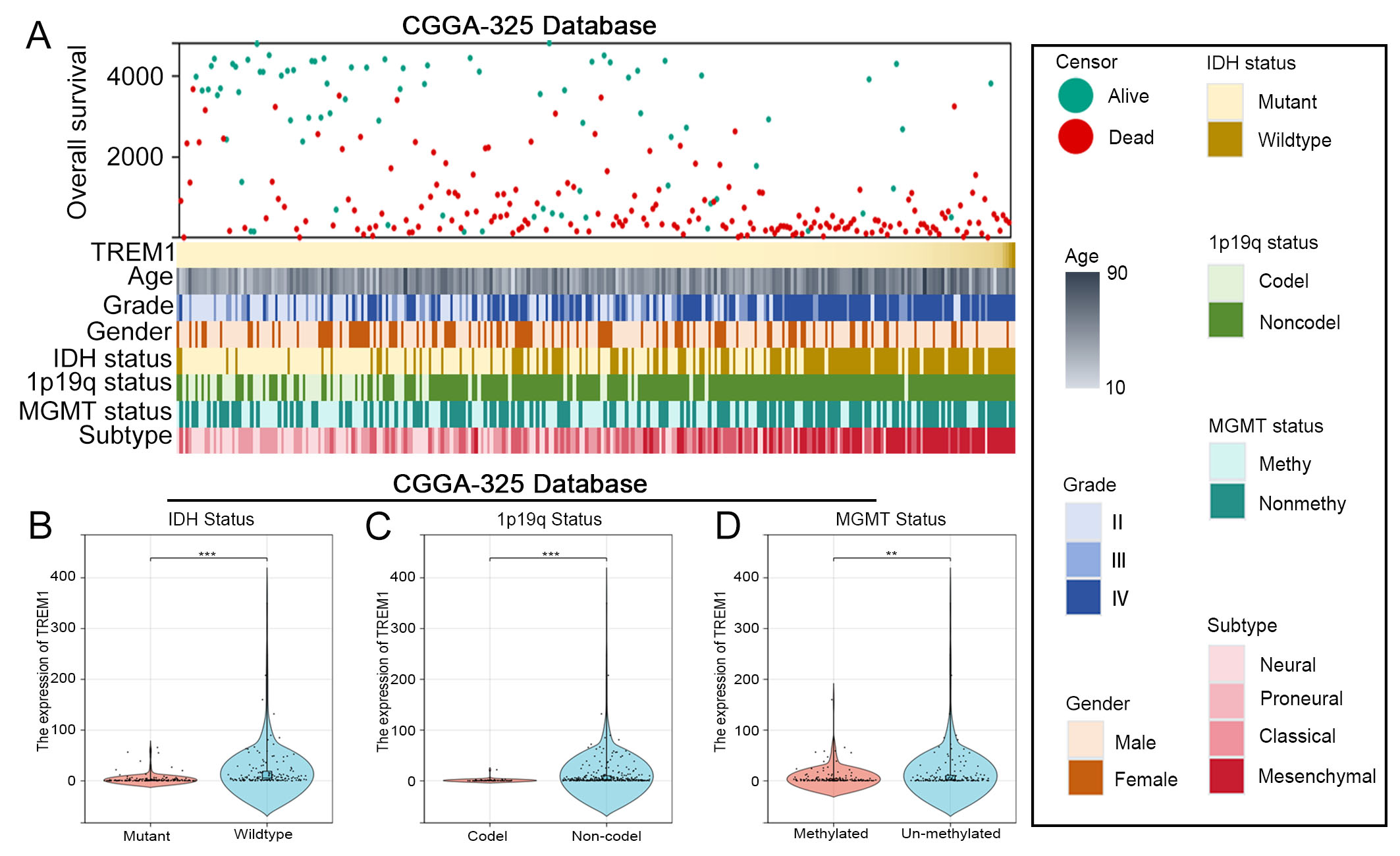


**Supplementary Figure S1:** Correlation of TREM1 with Clinical and Molecular Features in Glioma. A, Profile of TREM1 expression and clinical characteristics in the CGGA-325 database. B, Association between IDH mutation status and TREM1 expression. C, Association between 1p/19q co-deletion status and TREM1 expression. D, Association between MGMT promoter methylation status and TREM1 expression.

**Supplementary Figure S2**


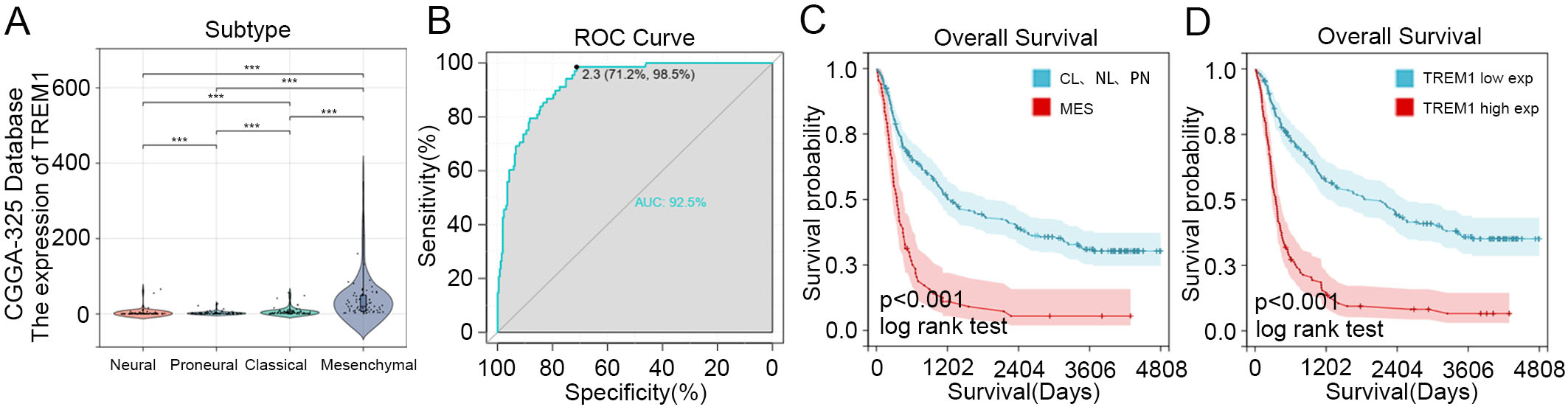


**Supplementary Figure S2:** Enrichment of TREM1 in the Mesenchymal Subtype of Glioma in the CGGA-325 database. A, TREM1 expression across glioma subtypes; B, Specific enrichment of TREM1 in the mesenchymal subtype; C, Prognosis of the mesenchymal subtype; D, Prognostic value of TREM1 expression.

**Supplementary Figure S3**


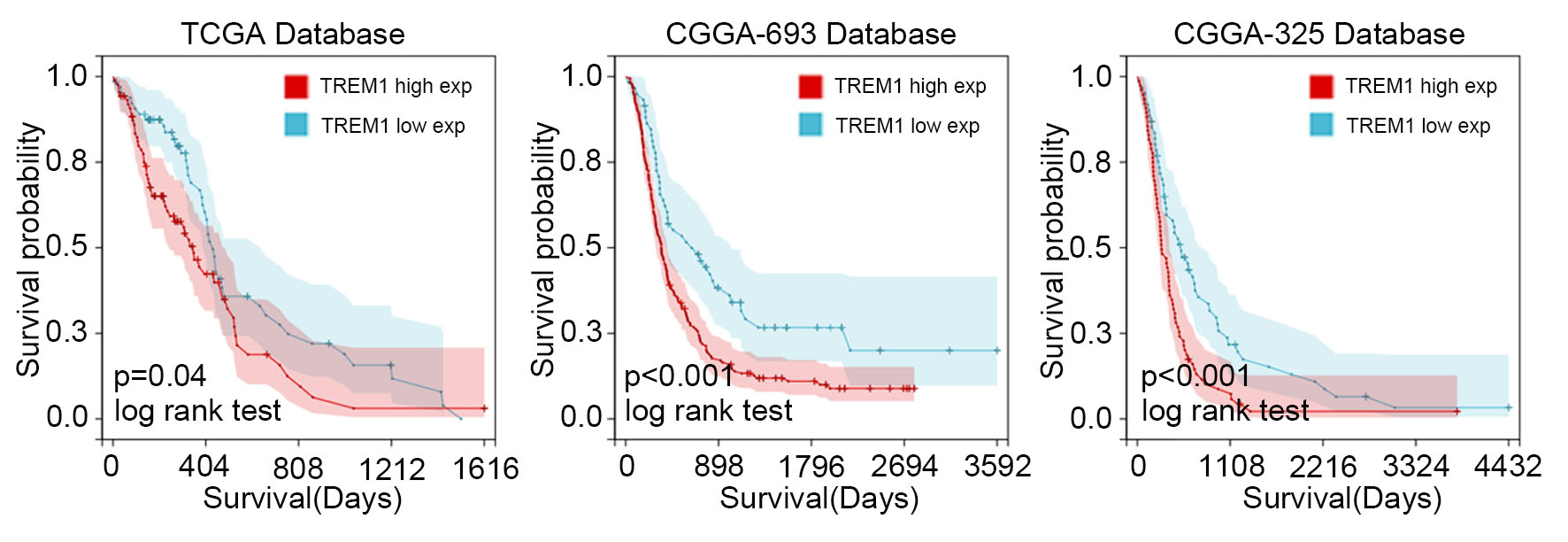


**Supplementary Figure S3:** Prognostic value of TREM1 expression in GBM patients.

**Supplementary Figure S4**


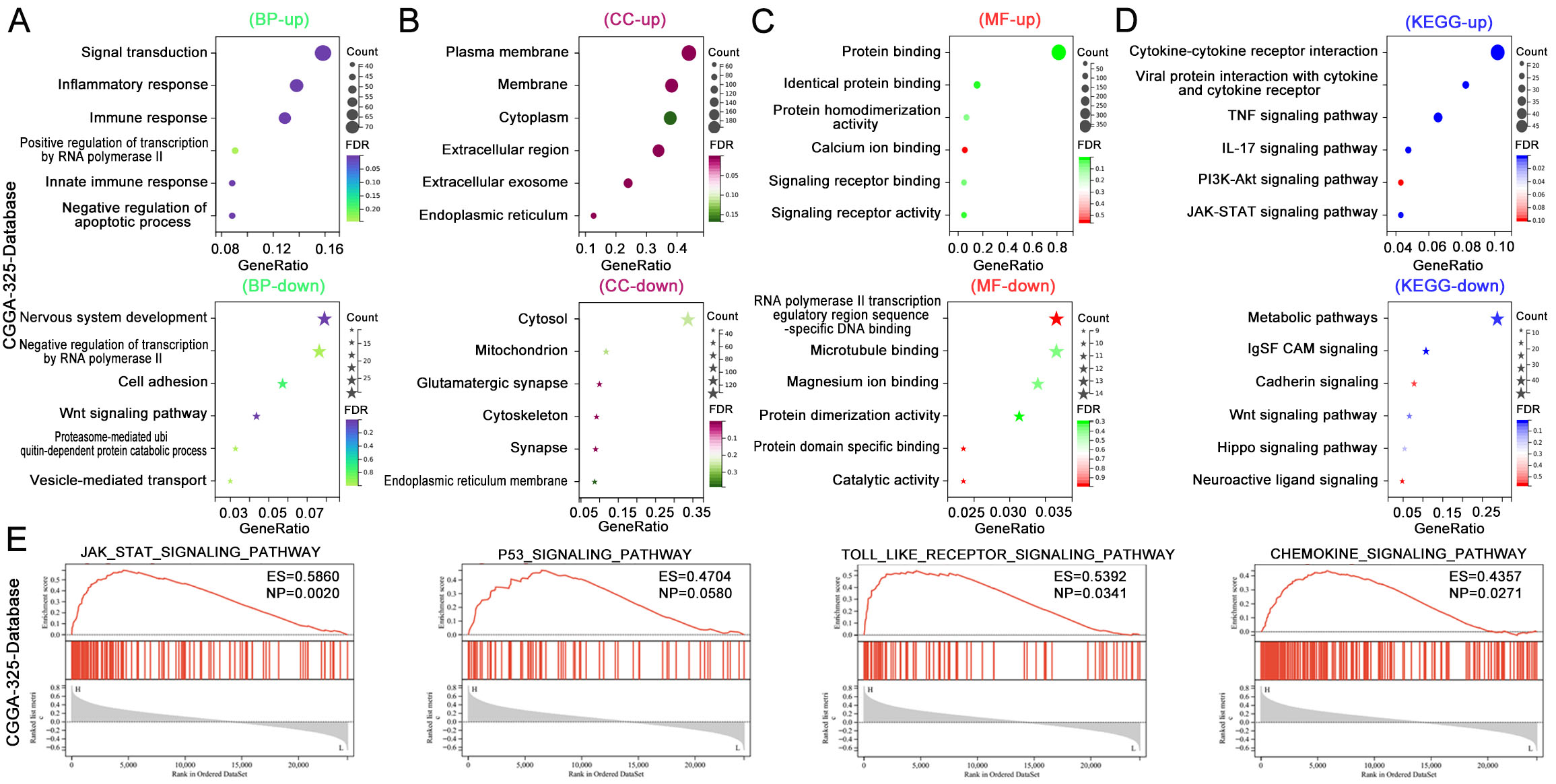


**Supplementary Figure S4:** GO and KEGG functional enrichment analysis of TREM1-related genes in the CGGA-325 database. A, GO analysis results for TREM1 co-expressed genes in BP; B, GO analysis results for TREM1 co-expressed genes in CC; C, GO analysis results for TREM1 co-expressed genes in MF; D, KEGG analysis results for TREM1 co-expressed genes; E, GSEA analysis of TREM1.

**Supplementary Figure S5**


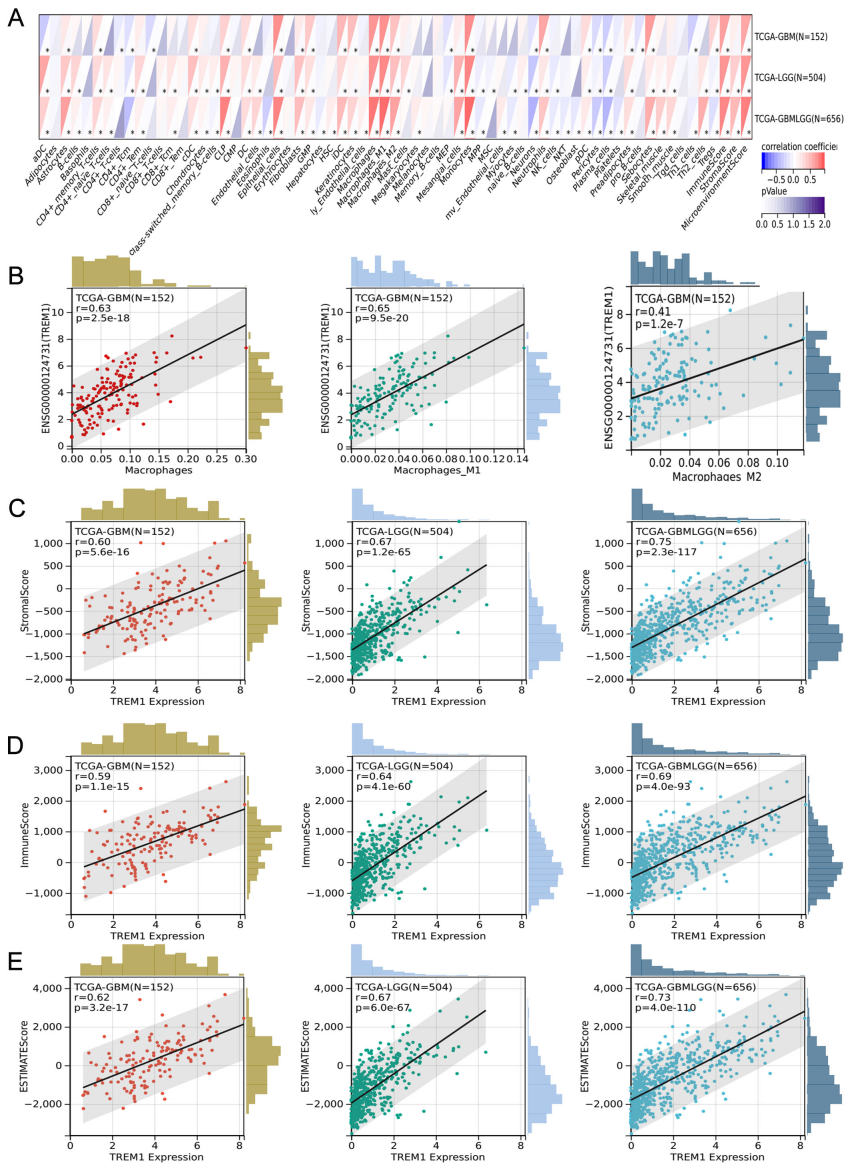


**Supplementary Figure S5:** Analysis of TREM1-Associated Immune Cells and Immune Infiltration in GBM. A, Examination of TREM1-Associated Immune Cells; B, Investigation of the Correlation Between TREM1 and Macrophages; C, Stromal Score Analysis of Immune Infiltration in GBM Patients; D, Immune Score Analysis of Immune Infiltration in GBM Patients; E, ESTIMATE Score Analysis of Immune Infiltration in GBM Patients.

**Supplementary Figure S6**


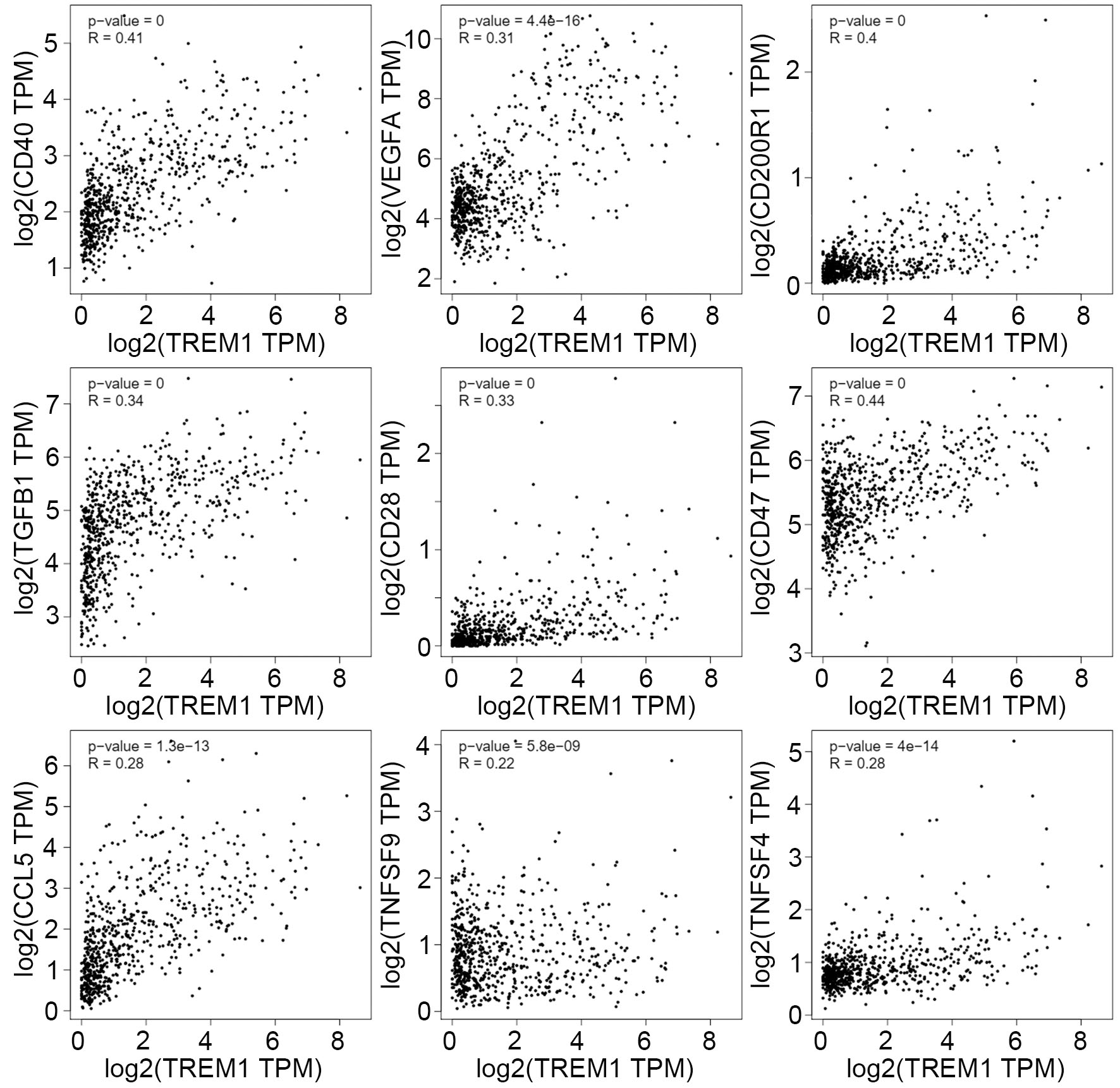


**Supplementary Figure S6:** Correlation analysis between TREM1 and immune checkpoints.

**Supplementary Figure S7**


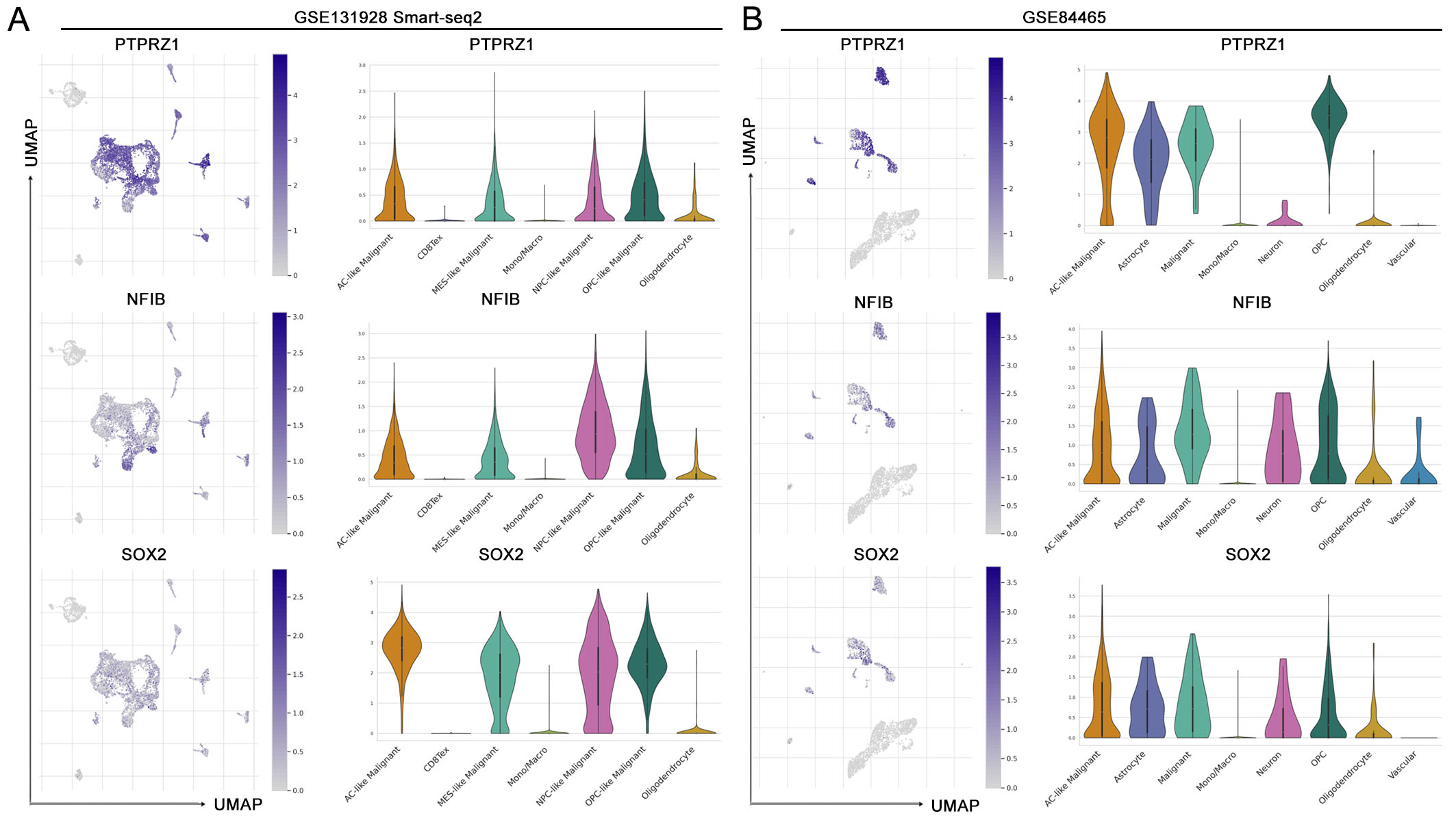


**Supplementary Figure S7:** Expression Features in the Single-Cell Atlas. A, Expression profiles of PTPRZ1, NFIB, and SOX2 in the GSE131928 Smart-seq2 dataset; B, Expression profiles of PTPRZZ1, NFIB, and SOX2 in the GSE84465 dataset.

**Supplementary Figure S8**


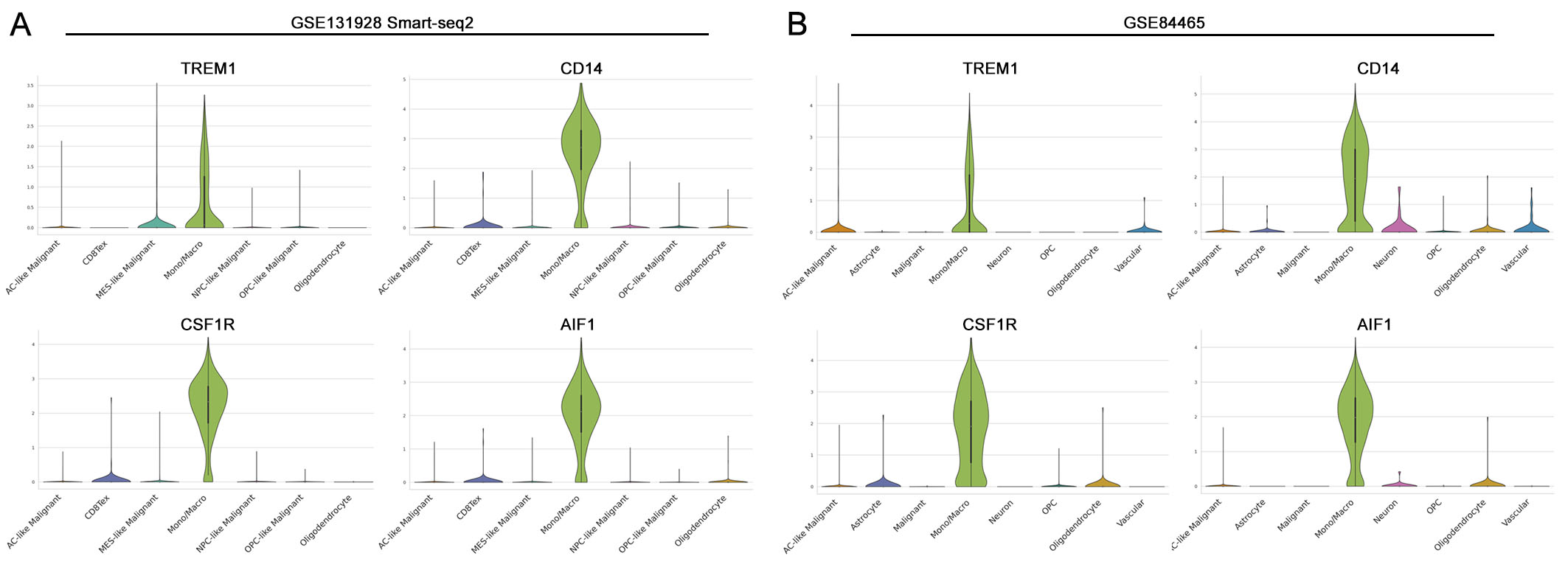


**Supplementary Figure S8:** Expression Features in the Single-Cell Atlas. A, Expression profiles of TREM1, CD14, CSF1R and SOX2 in the GSE131928 Smart-seq2 dataset; B, Expression profiles of TREM1, CD14, CSF1R and SOX2 in the GSE84465 dataset.

**Supplementary Figure S9**


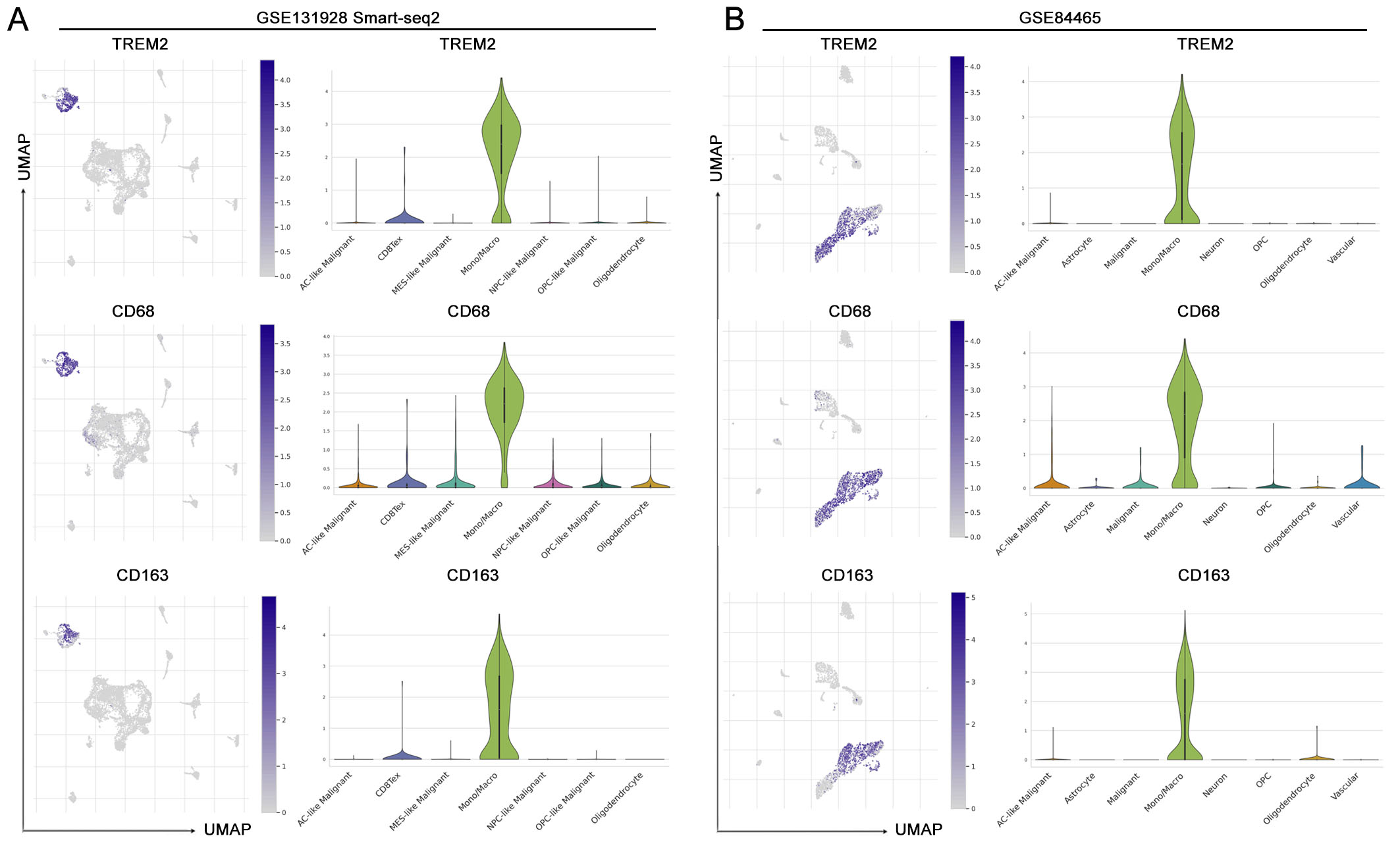


**Supplementary Figure S9:** Expression Features in the Single-Cell Atlas. A, Expression profiles of TREM2, CD68 and CD163 in the GSE131928 Smart-seq2 dataset; B, Expression profiles of TREM2, CD68 and CD163 in the GSE84465 dataset.

**Supplementary Figure S10**


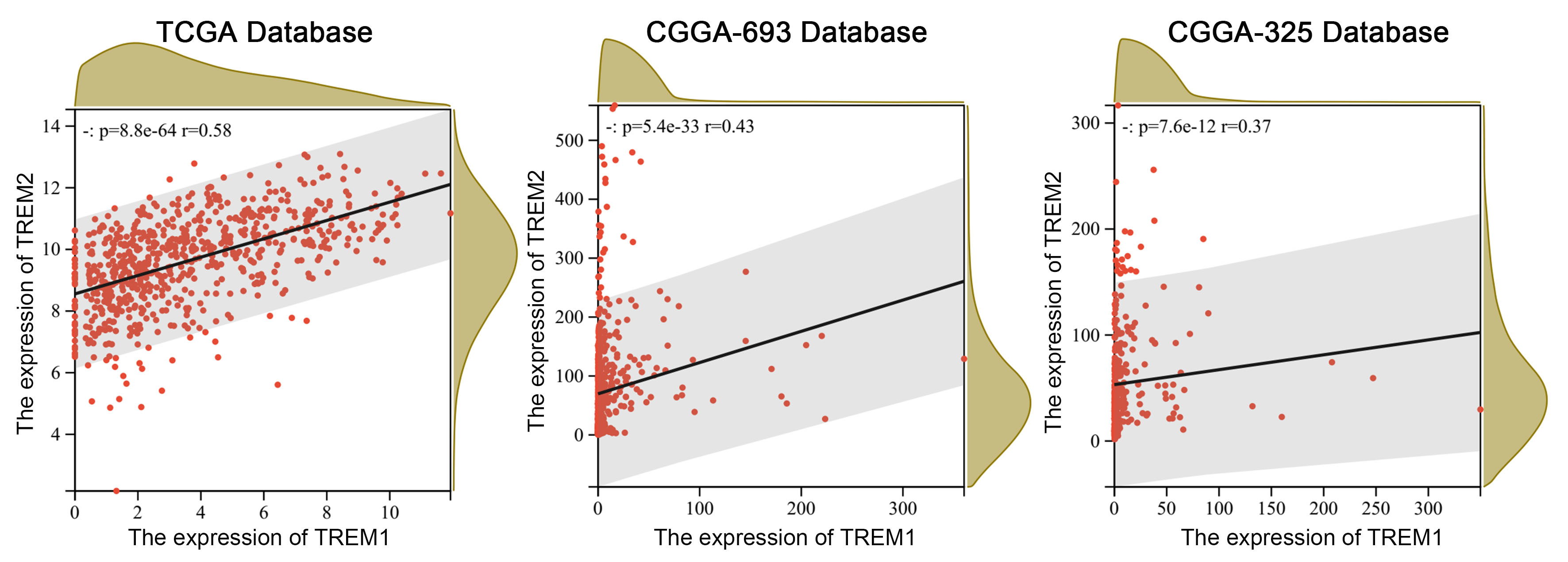


**Supplementary Figure S10:** Analysis of the correlation between TREM1 and TREM2.
